# CadmiLume: A Novel Smartphone-Based Bioluminescence Color-Tuning Assay and Biosensor for Cadmium and Heavy Metal Detection in Water Samples

**DOI:** 10.3390/mps8020033

**Published:** 2025-03-19

**Authors:** Vadim R. Viviani, Murilo S. Teixeira, Gabriel F. Pelentir

**Affiliations:** Departamento Física, Química e Matemática, Centro de Ciências e Tecnologias para Sustentabilidade (CCTS), Universidade Federal de São Carlos (UFSCar), Sorocaba 18052-780, SP, Brazil; murilo.sorbo@gmail.com (M.S.T.); gabrielpelentir@estudante.ufscar.br (G.F.P.)

**Keywords:** sensors, bioluminescence, luciferases

## Abstract

Heavy metal contamination of soil and water is a growing environmental concern, especially mercury, lead, and cadmium. Therefore, fast and reliable methodologies to assess contamination in the field are in demand. However, many methodologies require laborious, expensive, and cumbersome equipment that is not convenient for rapid field analysis. Mobile phone technology coupled with bioluminescent assays provides accessible *hands-on* alternatives that has already been shown to be feasible. Previously, we demonstrated that firefly luciferases can be harnessed as luminescence color-tuning sensors for toxic metals. An assay based on such a principle was already successfully applied for teaching biochemistry laboratory lessons, which demonstrates the effect of cadmium on enzyme function based on bioluminescence color change. For analytical detection of cadmium in water, here, we developed a novel bioluminescence assay using the cadmium-sensitive *Amydetes vivianii* firefly luciferase coupled with a cell phone provided with a program to quantify cadmium concentration based on luminescence color discrimination. The application has proven to be efficient with high precision between 0.10 and 2 mM of cadmium, being appliable to diluted water samples (0.1–2 µM) upon concentration and relying on reference cadmium standards values. The light emitted by the reference standards and samples in a dark box is captured by the smartphone’s camera, which, using computer vision, automatically quantifies cadmium according to the RGB color. CadmiLume is a simple and easy luminescent enzymatic biosensor for cadmium contamination in water samples, which instantaneously can provide results with the convenience of a smartphone in the palm of one’s hands.

## 1. Introduction

Heavy metal contamination of water and soils constitutes a major environmental concern [1]. Cadmium, which is released by batteries and electric industry products, is a very toxic metal associated with chronic and severe diseases [1]. Metals such as cadmium and mercury bind to non-specific sites in proteins and enzymes, affecting their function and, therefore, cell metabolism, causing genotoxicity [1,2].

Several colorimetric and photometric analytical kits, which provide results within 15–30 min and display sensitivities in the nanomolar range, are currently commercially available [3,4,5].

Fluorescent biosensors have been also developed for cadmium and other metal detection [6,7,8,9,10,11,12,13,14,15]. Most fluorescent sensors are based on intensity, the so-called *light on/off* sensors, which report the presence of metals by increasing or decreasing the fluorescence intensities, or ratiometric, which report the ratio of excitation or fluorescence intensities at different wavelengths [6]. The ratiometric sensors are more specific and devoid of drawbacks, such as the concentration dependency of the probe. Whole-cell fluorescent biosensors for cadmium based on inducible promoters, CadC and CadR, expressing GFP or its variants, like Cherry, with sensitivities ranging from 1 to 400 µM, were constructed [13,14,15]. Ratiometric biosensors based on FRET from ECFP and cpVenus, with sensitivities of ~250 nm, were also developed [15]. Whereas fluorescence sensors are popular and quite sensitive, they have disadvantages, including the need for an external irradiation source, phototoxicity, and problems associated with the auto-absorption and autofluorescence of the samples, which reduce the signal/noise ratio and often need the use of cumbersome equipment, which make their effective use in the field unreliable. Furthermore, whole-cell *light on* biosensors based on inducible promoters may require several hours to obtain results. Therefore, easy *hands-on*, and accessible biosensors are in demand. Furthermore, educational strategies are also needed to increase public awareness regarding the toxicity of these metals, especially in biological and environmental sciences university courses, which would benefit from easy laboratory lessons that demonstrate the subtle effects of these metals on protein function, especially enzymes.

Bioluminescent sensors offer alternatives for heavy metal detection [16,17,18,19,20,21,22,23]. As in the case of fluorescent sensors, the bioluminescent sensors can be *light on/off* [16,17,18] or ratiometric. *Light off* biosensors based on bioluminescent bacteria and their Lux operon were the first to be used and are still quite popular [16] but lack specificity for different metals. Similarly, *light off* biosensors based on beetle luciferases were also attempted [17]. *Light on* whole-cell BL sensors for cadmium and mercury have been proposed [18,19], but their effective use has not been accomplished yet. Ratiometric BL sensors, which are based on BRET, have been also developed and used mostly for calcium-sensing [20,21,22]. In the past years, bioluminescent assays were also associated with smartphone technology to provide *hands-on* field biosensors for *point-of-care* medicine and environmental analysis [23].

Firefly luciferases, the enzymes that are responsible for the ATP-dependent oxidation of a benzothiazolic luciferin-yielding bioluminescence [24], were shown to be very useful for several bioanalytical applications, including ATP assays and reporter genes, and they are used in biosensors and bioimaging [23,25,26]. More recently, they were also shown to be potentially useful as ratiometric color-tuning indicators of intracellular pH [27,28] and as sensing proteins for heavy metals such as zinc, mercury, cadmium, and lead [29]. However, many firefly luciferases are rather non-specific for heavy metal sensitivity [28].

The luciferase of *Amydetes vivianii* was shown to be especially selective for cadmium [30], changing the bioluminescence color from green to orange at high concentrations of cadmium. This luciferase has already been successfully used in biochemistry laboratory lessons in undergraduate university courses to demonstrate the effect of cadmium on protein function [31]. Based on such principle, here, we developed a new smartphone-based luminescent biosensor and Elisa-based bioluminescence assay, which allows us to quickly quantify cadmium in water samples based on colorimetric analysis of luciferase elicited luminescence for field biosensing and for university teaching courses.

## 2. Experimental Design

### 2.1. Materials

**Reagents**. D-luciferin potassium salt was kindly provided by René M. Hiensch (Resem BV, Lijnden, The Netherlands). ATP was purchased from SIGMA. All other reagents were of the highest quality.

**Luciferase purification**. The *Amydetes vivianii* firefly luciferase (AmyLuc) used in this protocol was expressed in bacteria and purified by nickel–agarose chromatography according to a previously published method [30]. The luciferase was provided at a concentration of 0.5–1 mg/mL in a 0.10 M Tris-HCl buffer at pH 8.0 supplemented with 15% glycerol, and it was stable for at least 2 months when stored at 4° C.

**Cadmium standards.** Cadmium sulfate stock solutions were prepared in pure MilliQ water for the following concentrations: 20 mM, 15 mM, 10 mM, 7.5 mM, 5 mM, 2.5 mM, and 1 mM. These stock solutions as well as water (10 µL) are pipetted in the wells of an Elisa plate before adding the assay solution.

**Assay solution.** The dilution solution consists of 0.10 M Tris-HCl pH 8.0, 10% glycerol, 0.5 mM luciferin, 2 mM ATP, and 4 mM MgSO_4_ (final concentrations). Ideally, prepare the dilution solution, cool it on ice, and just before the assay, add D-luciferin and MgATP. This assay solution can be divided into aliquots and stored at −20 °C, shielded from light with aluminum foil for 1 month. Just before the assay, add 50 µL of AmyLuc (0.5 mg/mL) to 950 µL of assay solution, keep it on ice, and aliquot 90 µL of this mix to the Elisa plate wells.

**Sample pre-treatment and concentration**. To detect the presence of metal traces in diluted water samples, the samples can be concentrated 10–10.000times by evaporation at 75–85 °C or even dried up and resuspended in a 0.10 M Tris-HCl buffer at pH 8.0. Usually, a 10–1000 times concentration is recommended. Interferences caused by concentration are unlikely to affect pH, as the assay solution is strongly buffered.

### 2.2. Equipment

**Smartphone**. The smartphone used to take pictures and image analysis was a Samsung Galaxy model S10Plus, which is representative of a modern CCD-based cell phone, with enough sensitivity to obtain pictures in dark environments. It features an MP camera with a variable aperture and an ISO range of up to 3200. These specifications allow for high-sensitivity imaging of bioluminescent samples, ensuring that even weak luminescence can be captured accurately. Other cellphones were also successfully used.

## 3. Procedure


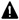
 The protocol consists of mixing AmyLuc purified luciferase to the assay solution on ice, followed by pipetting 90 µL of the complete assay solution to Elisa plate wells containing 10 µL of cadmium sulfate standards or water samples. Then, photography is obtained and analysis of luminescence color with a smartphone CCD camera provided with a proprietary image analysis program is performed.

### 3.1. Bioluminescent Assay

In a row of 96 Elisa plate wells, add 10 µL of pure water as a control and the cadmium sulfate standards (0.1, 0.25, 0.5, 0.6, 0.8, 1.0, 1.5, 2 mM). In another row, add the supposedly contaminated water samples to each well. To each well of the Elisa 96 well plate containing the 10 µL of cadmium standards or samples, add 90 µL of freshly prepared ice-cold assay solution (AmyLuc, D-luciferin, ATP, MgSO_4_). The reactions are left at room temperature (20–25 °C) for 5–10 min in a dark box.


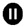
 **PAUSE STEP**. The maximum luminescence intensity and color change that can be visually and photographically detected occur between 5 and 10 min after mixing the reagents at 22 °C. If the assay is performed at lower environmental temperatures, the time can be increased up to 15 min, whereas at higher temperatures the time can be shorter. In any case, do not exceed 30 °C!

### 3.2. Smartphone Photographic Detection

**Figure 1:**
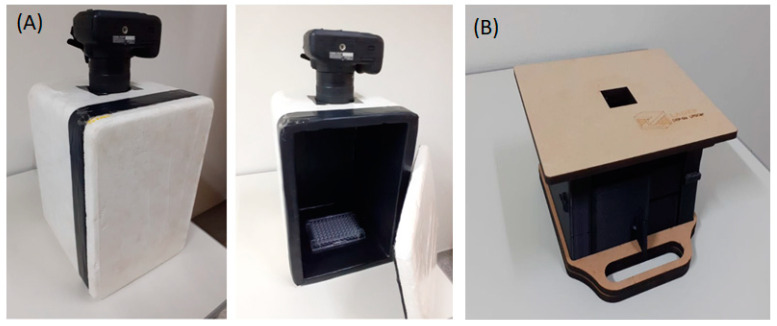
Dark boxes used for photographic detection using a smartphone: (**A**) a homemade dark box for a photographic camera/cell phone showing the Elisa plate inside; (**B**) smartphone-designed dark box.

After 5–10 min, the photographic image is obtained by positioning a standard smartphone CCD camera in a custom-adapted dark box for cell phones (Figure 1), with the detector placed at least 6 cm away from the samples in order to take a focused image of the field containing the standards and samples. To maintain consistency and minimize external light interference, all images are taken inside a custom-built dark box, preventing ambient light interference (Figure 1). The camera operates in manual mode, where ISO, exposure time, and white balance settings are adjusted by the user to ensure optimal luminescence detection. If necessary, the CCD camera shall be used in night mode (or pro mode with long exposure settings) to enhance sensitivity, capturing low-intensity emissions with greater precision (>1000 ISO).

#### 3.2.1. Smartphone Application

The developed application quantifies cadmium concentrations in environmental samples through luminescence and colorimetric analysis, utilizing image processing via OpenCV, an open-source computer vision library that enables advanced image analysis, including object detection, segmentation, and color processing. It guides users in capturing and analyzing images, providing results in both visual and numerical formats. The process begins with image capture using controlled lighting to ensure accuracy. The app identifies and aligns sample wells, applying segmentation techniques to isolate areas of interest. Colorimetric analysis is then performed by extracting RGB values, converting them to chromatic coordinates, and quantifying cadmium concentration based on reference standards.

#### 3.2.2. Image Analysis and Cadmium Quantification

**Figure 2:**
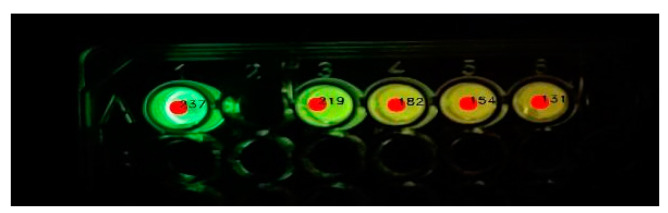
Area selection of the luminescent samples/standards for color analysis.

**Figure 3:**
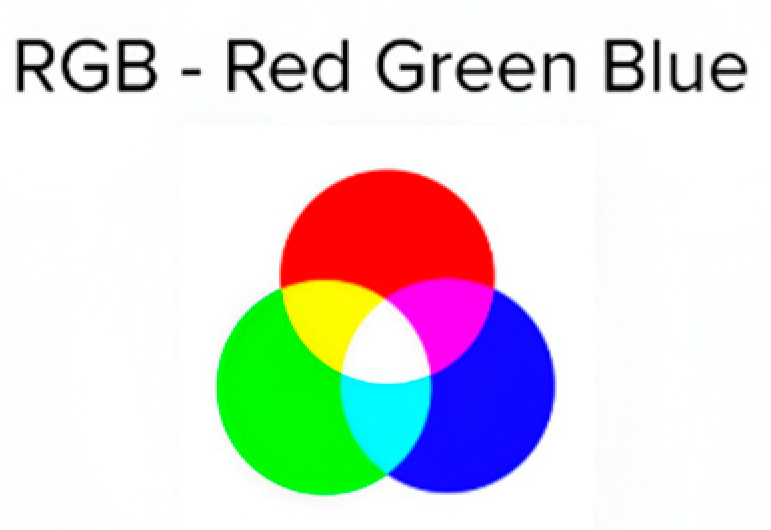
Colors used in the RGB analysis.

The application processes the image automatically, detecting sample wells and extracting colorimetric information to estimate cadmium concentration. Using OpenCV’s thresholding and contour detection methods, areas of interest are isolated to minimize external interference (Figure 2). Extracted RGB values are then converted into normalized chromatic coordinates, reducing lighting variations (Figure 3). According to our previous studies [32], also shown in the Supplemental Material (Figure S1), there is a linear relationship between cadmium concentration and the ratio of green and red light intensities. A proprietary mathematical model (S2) maps these values onto a concentration scale, correlating luminescence intensity with cadmium levels.

#### 3.2.3. User Interface and Result Display

**Figure 4:**
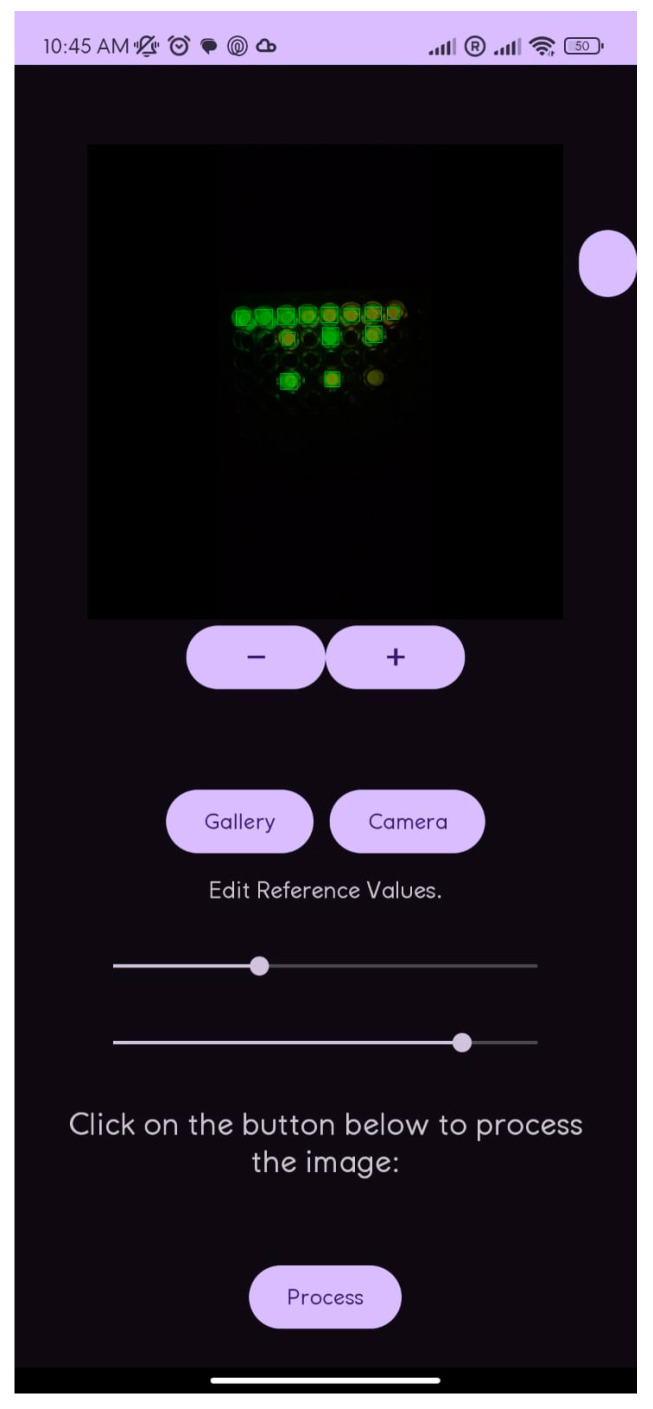
Screen of cell phone analysis.

The application displays the analyzed image with annotations indicating detected concentrations for each well, providing both a visual and numerical representation of the results. A table presents values in millimolars (mM) and is organized by sample position for easy recording and comparison. The interface includes controls for capturing and selecting images, adjusting analysis parameters, and tools for image rotation and zooming (Figure 4), ensuring ease of use in obtaining reliable results.

## 4. Results

**Figure 5:**
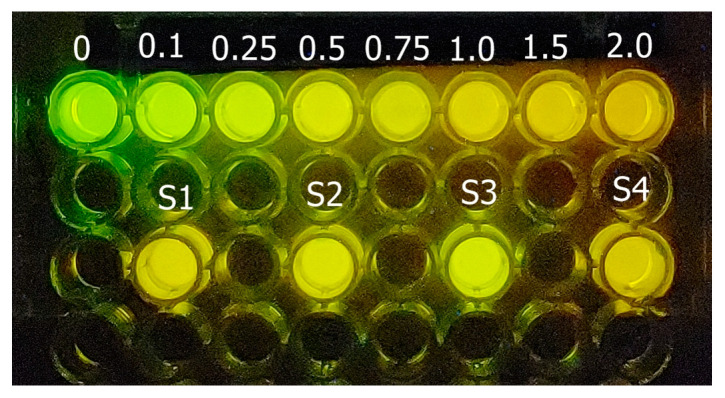
Cadmium luminescence color assay. The standard cadmium concentrations (0–2 mM) are in the upper row and there are 4 different cadmium samples (S1–S4) in the lower row. Upon using the cell phone imaging application, a linear relationship was first obtained between the cadmium concentration of the standards and the ratio of green and red color, whereby the green color decreases as the cadmium concentration increases.

**Figure 6:**
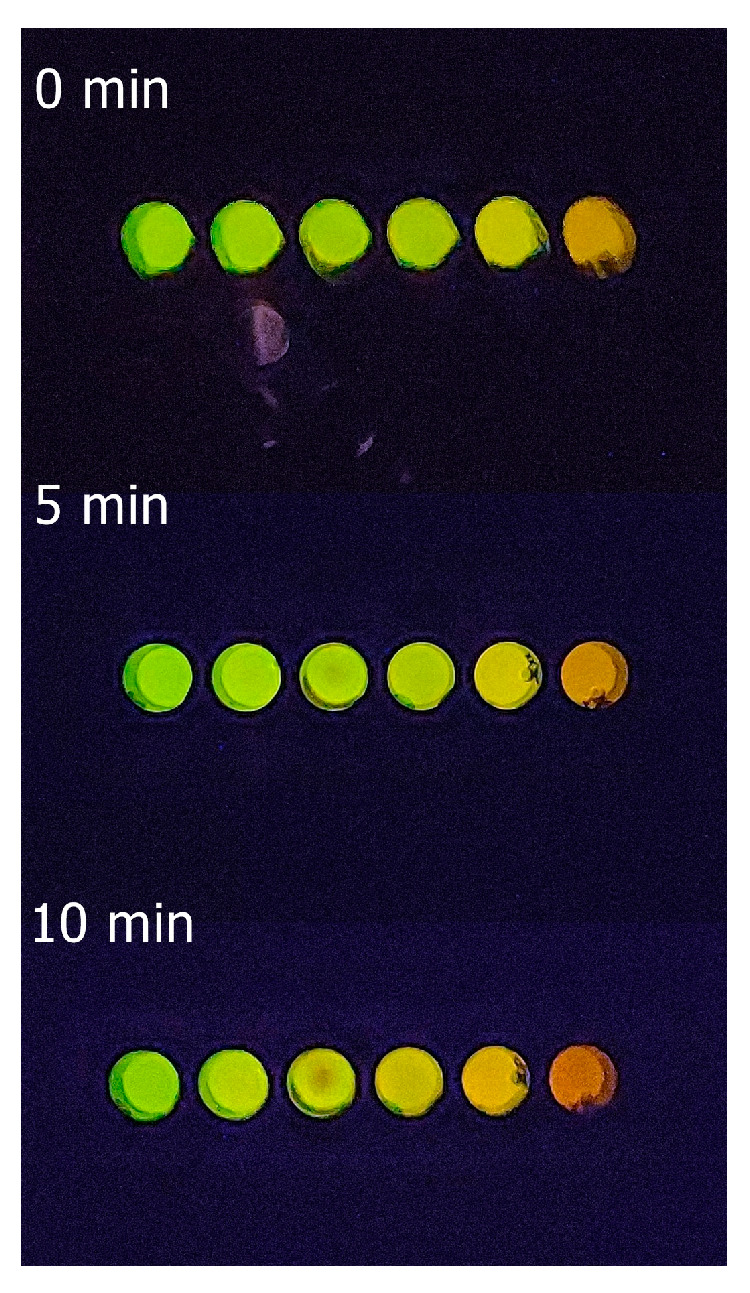
Elisa plate showing the bioluminescence color variation of standard cadmium solutions (references) at different times (0–10 min) after mixing the reagents at 22 °C.

As one may see, after mixing the reagents, there is a clear and intense luminescence color change from green for the control reaction without cadmium (Figure 5), to yellow-green (0.1–0.25 mM), yellow (0.5–1 mM), and orange (1.5–2 mM) as the cadmium concentration increases. The change of luminescence color is more evident after 5–10 min of mixing the reagents at 22 °C (Figure 6); therefore, we standardized it to 5 min. Visually, depending on the cadmium concentration (Figure 5), the samples show that the color approaches those of the standards.

**Figure 7:**
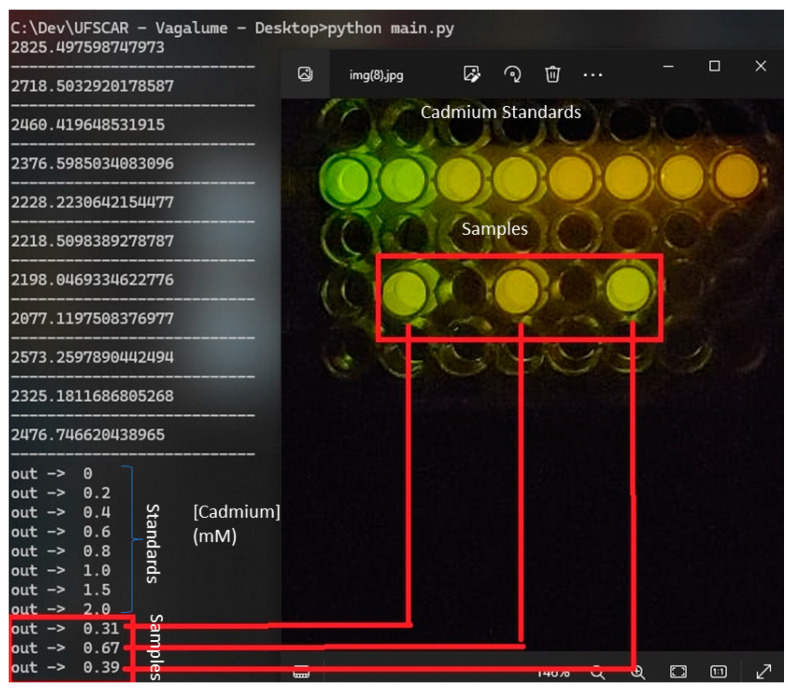
Results of the smartphone bioluminescence color analysis of the cadmium-containing samples.

Figure 7 shows the values of cadmium concentrations of one experiment obtained for three samples based on the reference cadmium concentrations. The processed results (Figure 7) were based on the standard cadmium concentrations varying from 0.10 to 2 mM (reference values). Based on the cell phone analysis of bioluminescence colors, the estimated concentration values for the samples containing cadmium corresponded to the expected values, with an average standard deviation from the expected values of ±6.2% (variation = 1–22%; sampling: 10) in relation to the expected values. The use of the application allows us to achieve a color distinction that is not easily detectable with the naked eye. Because fresh and sea water have natural cadmium concentrations ranging from 24 to 240 nM, respectively and the toxic cadmium concentration limits for water samples allowed by sanitary agencies (24 nM) are usually much lower than the detectable cadmium concentration using this methodology (0.1–2 mM), the same procedure was then applied to very diluted water samples containing cadmium (1–20 µM) that were 100 times concentrated by evaporation, giving off the expected values.

## 5. Discussion

The natural cadmium concentrations found in natural water samples range from 24 to 240 nM. Most current detection methods are sensitive enough to detect higher concentrations. Current commercial kits for cadmium detection, which include photometric and strip-based colorimetric tests, display sensitivities ranging from 0.02 to 1 ppm (0.02–1 mg/L).

In the case of luminescent cadmium sensors, most of them are fluorescent. Some of them are whole-cell biosensors based on *light on* inducible *Cad*C and *Cad*R cadmium-binding proteins and fluorescent proteins, such as GFP or *Cherry*, or ratiometric biosensors based on FRET between ECFP and *Venus* mediated by *Cad*R [13], displaying sensitivities ranging from 0.1 and 400 µM. However, fluorescent biosensors have the usual drawbacks associated with fluorescence, such as the need for excitation sources, phototoxicity, and autofluorescence.

Bioluminescent biosensors based on bacterial luciferase and Lux operon and *znt*A promoters were also constructed. The *light off* biosensors based on bioluminescent bacteria were the first to be used and are still quite popular; however, they lack the specificity of most heavy metals, and other substances affect bioluminescent intensity [32]. On the other hand, despite being more specific and more sensitive, detecting up to 0.2–1 ppm of cadmium, most whole cell *light on* biosensors based on inducible promoters, such as *Cad*C and *znt*A, are more laborious and require long procedures and induction times taking several hours (sometimes up to 17 h) [33,34].

Thus, although our cadmium smartphone-based biosensing assay has a sensitivity that is slightly lower (100 to 2000 µM or ~20–400 ppm) than some fluorescent sensors, as an enzymatic biosensor, it is faster and avoids the problems associated with fluorescence. Furthermore, it is simpler, requiring just a single luciferase and not two components as required for FRET and BRET systems.

However, when comparing the sensitivity of our detection method (~20–400 ppm) with the commercially available kits (0.025–1 ppm), our method is still ~20–400 times less sensitive. The lower sensitivity can be circumvented by concentrating 10–1000 times the water samples, which can be easily attained after centrifugation to remove particulate material, and drying the samples at 75–95 °C for a few hours, depending on the sample volume. An issue to be considered when concentrating on water samples, however, is the matrix effect with the concentration of compounds, which may interfere with the luciferase assay. Considering that the assay solution is strongly buffered, it is expected that changes in pH, which may arise from concentrating on the samples, will not be a major issue. Additional studies could be necessary to verify whether the concentration of other compounds present in the complex matrix of the samples may affect the luciferase assay. Another possible disadvantage of this assay is that this luciferase, although more selective to cadmium, also displays some sensitivity to mercury [30]. However, mercury has a higher inhibiting effect on luciferase activity than cadmium. Thus, in some cases, this luciferase assay could be also used to detect mercury or both metals. The main advantages of this assay are the fact that it is a simple enzymatic biosensor, devoid of complexities of whole-cell biosensors, and its rapidity, requiring just 5 min upon availability of the reagents and cell phone.

Overall, this smartphone-based method has the advantage of its practicality and rapidity, providing an easy *hands-on* biosensor and assay, which allows us to readily obtain results in the field that can be exported to an analysis center. As we already demonstrated, this assay is very interesting when demonstrating the subtle effects of heavy metals in protein/enzyme function in biochemistry laboratory lessons for biological and environmental sciences undergraduate courses [31]. Another possibility showing the versatility of this assay is the substitution of AmyLuc by other metal-sensitive or pH-sensitive firefly luciferases to detect other metals, such as mercury, zinc, and lead, or to monitor intracellular pH changes inside cells.

## 6. Concluding Remarks

- Here, we developed an innovative smartphone-based luminescent color assay for cadmium detection and quantification in water samples, which uses a novel cadmium-sensing luciferase, AmyLuc.
- The method provides a fast cell-free *hands-on* ratiometric biosensor for the field, which, based on the reference cadmium concentration values, can detect cadmium in the range from 20 to 400 ppm.
- The sensitivity of the assay could be extended to 0.02–0.4 ppm upon sample concentration.
- The cellphone-based assay instantly provides images and analysis of numerous samples in the field, which can be exported via telecommunication without the need for expensive and inconvenient equipment.
- Based on the same principle, this assay can also be easily adapted to luminescence colorimetric detection of other analytes, including other heavy metals, such as mercury, depending on the selected metal-sensitive luciferase or pH using pH-sensitive firefly luciferases.

## 7. Patents

This work refers to patent applications BR512024003594-0; BR 1020200003003; and BR 10 20210166410.

## Data Availability

Data will be available upon request. The program application is not available yet.

## Supplementary Materials

The following supporting information can be downloaded at https://www.mdpi.com/article/10.3390/mps8020033/s1, Figure S1: Effect of cadmium concentrations on the in vitro bioluminescence spectrum of Amydetes luciferase: (A) effect of different concentrations of CdSO4 on the bioluminescence spectra; (B) effect of different concentration of CdSO4 on the ratio of bioluminescence intensities at λgreen and λred (R = Ired/Igreen). From Pelentir et al., 2020 [28]; S2: Mathematical process used to evaluate the color of each sample.
