# Supplementary material for: CadmiLume: A Novel Smartphone-Based Bioluminescence Color-Tuning Assay and Biosensor for Cadmium and Heavy Metal Detection in Water Samples
Source: Methods Protoc. 2025 Mar 19;8(2):33. doi: 10.3390/mps8020033 (PMC11932295; doi:10.3390/mps8020033)
Supplement: Supplementary file 1 [file mps-08-00033-s001.zip › mps-3444700-supplementary.pdf]

8. Supplementary Materials:

S1. Effect of cadmium concentration on bioluminescence spectra and ratiometric calibration curve

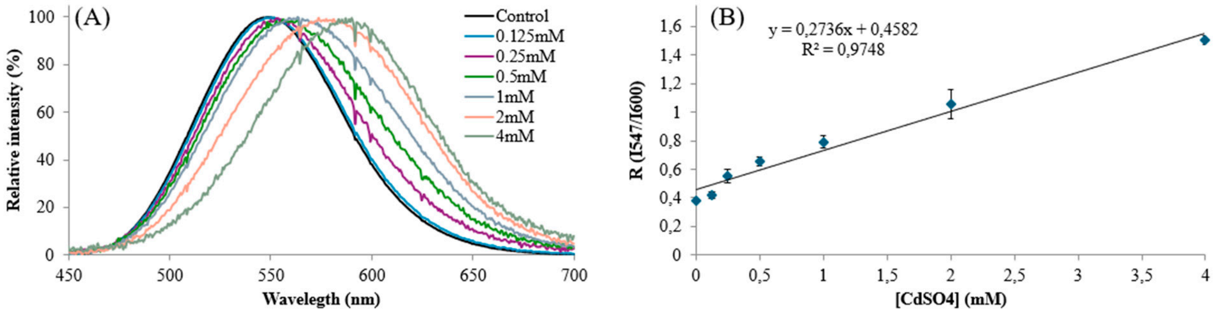

Figure S1. Effect of cadmium concentrations on the *in vitro* bioluminescence spectrum of *Amydetes* luciferase: (A) effect of different concentrations of $CdSO_4$ on the bioluminescence spectra; (B) effect of different concentration of $CdSO_4$ on the ratio of bioluminescence intensities at λ_{green} and λ_{red} ($R = I_{red}/I_{green}$). From Pelentir et al., 2020 [28]

S2. Description of the mathematical process used to evaluate the color of each sample:

Averaging Color Channels:

The code extracts a 30x30 pixel region around a given point and computes the mean values of each color channel (Blue, Green, Red).

Color Space Conversion (BGR to XYZ-like Coordinates):

Using the average channel values, a linear transformation is applied:

$$X = (-0.14282 \cdot r) + (1.54924 \cdot g) + (-0.95641 \cdot b)$$

$$Y = (-0.32466 \cdot r) + (1.57837 \cdot g) + (-0.73191 \cdot b)$$

$$Z = (-0.68202 \cdot r) + (0.77073 \cdot g) + (0.56332 \cdot b)$$

This step converts the mean RGB values into a set of coordinates (X, Y, Z) related to the CIE 1931 XYZ color space or a similar intermediate color representation.

Normalization to Chromaticity Coordinates (x,y):

Next, chromaticity coordinates are derived:

$$X_{norm} = \frac{X}{X+Y+Z} \qquad Y_{norm} = \frac{Y}{X+Y+Z}$$

These normalized values represent the color's position on the standard 2D chromaticity diagram.

McCamy's Approximation for Correlated Color Temperature (CCT):

Using the chromaticity coordinates, the code computes:

$$n = \frac{X_{norm} - 0.332}{0.1858 - Y_{norm}}$$

With n calculated, it applies an empirical formula (McCamy's formula) to estimate the correlated color temperature:

$$CCT = 449n^3 + 3525n^2 + 6823.3n + 5520.33$$

Therefore, the calculation takes local average color values from the image (in RGB), converting them into a chromaticity space, and then using a known approximation formula to derive an approximate color temperature (CCT) from those chromaticity coordinates. This is a standard method in color science to estimate the "white point" or color temperature from measured color values.
